# Supplementary figures and images for: A light-inducible protein clustering system for in vivo analysis of α-synuclein aggregation in Parkinson disease
Source: PLoS Biol. 2022 Mar 9;20(3):e3001578. doi: 10.1371/journal.pbio.3001578 (PMC8936469; doi:10.1371/journal.pbio.3001578)

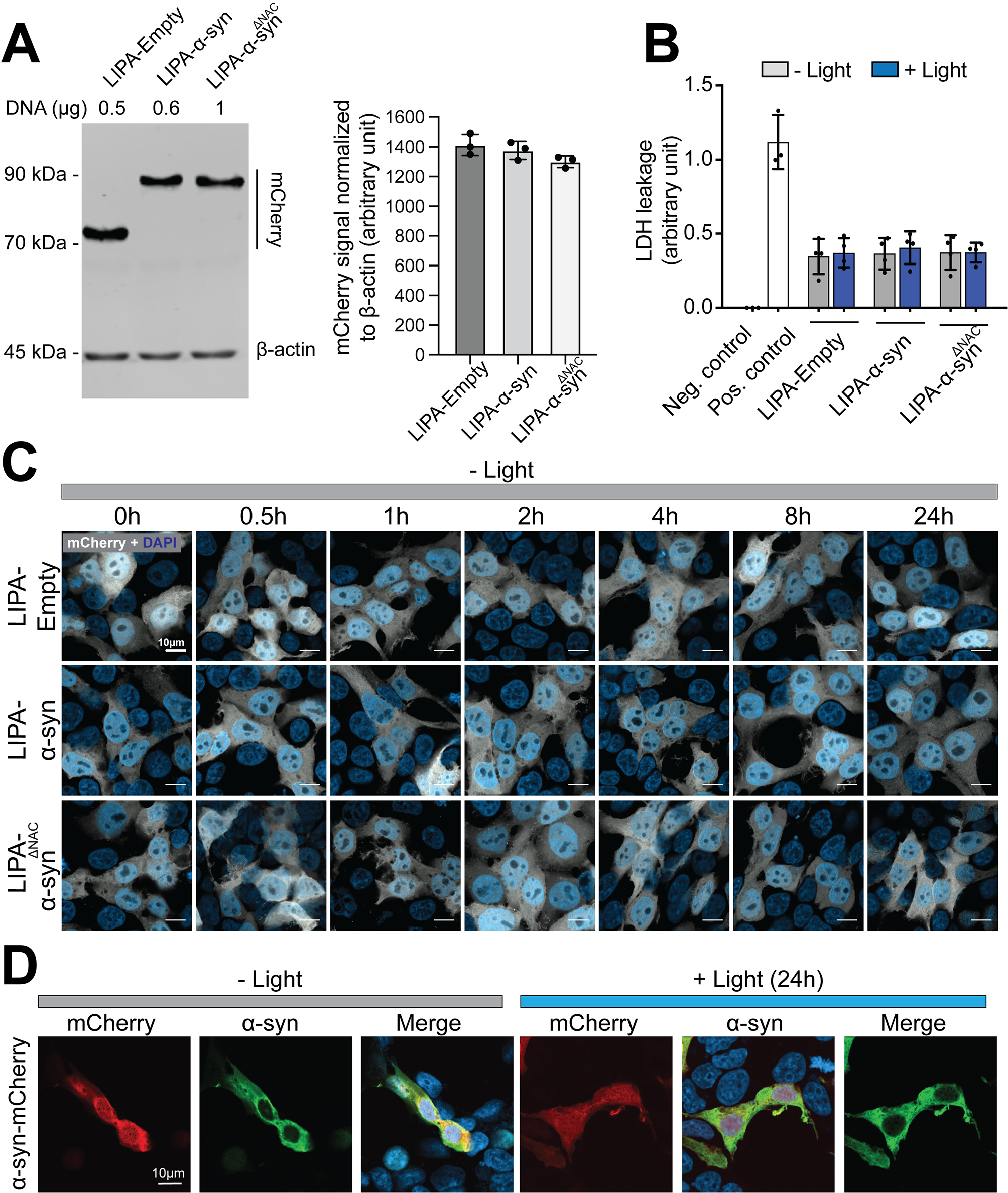

Supplement: S1 Fig — (A) Western blot and quantification of the protein levels (mCherry) of the different LIPA constructs showing similar expression levels after DNA normalization during the transient transfection (n = 3). The data are presented as the means ± SEM. (B) Cell toxicity assay assessed by quantifying the extracellular release of cytosolic LDH from HEK-293T cells overexpressing LIPA constructs exposed (blue histograms) or not (gray histograms) to 24 hours of continuous blue light stimulation at 0.8 mW/mm2 (n = 4). The data are presented as the means ± SEM. (C) In the absence of blue light stimulation, LIPA constructs exhibit diffuse cytosolic expression without detectable protein aggregates (n = 5) (scale bar = 10 μm). (D) HEK-293T cells overexpressing α-syn-mCherry, exposed or not to the blue light, did not display inclusion formation (n = 3) (scale bar = 10 μm). The underlying data for (A) and (B) can be found in S1 Data. LDH, lactate dehydrogenase; LIPA, light-inducible protein aggregation. (TIF) [file pbio.3001578.s001.tif]

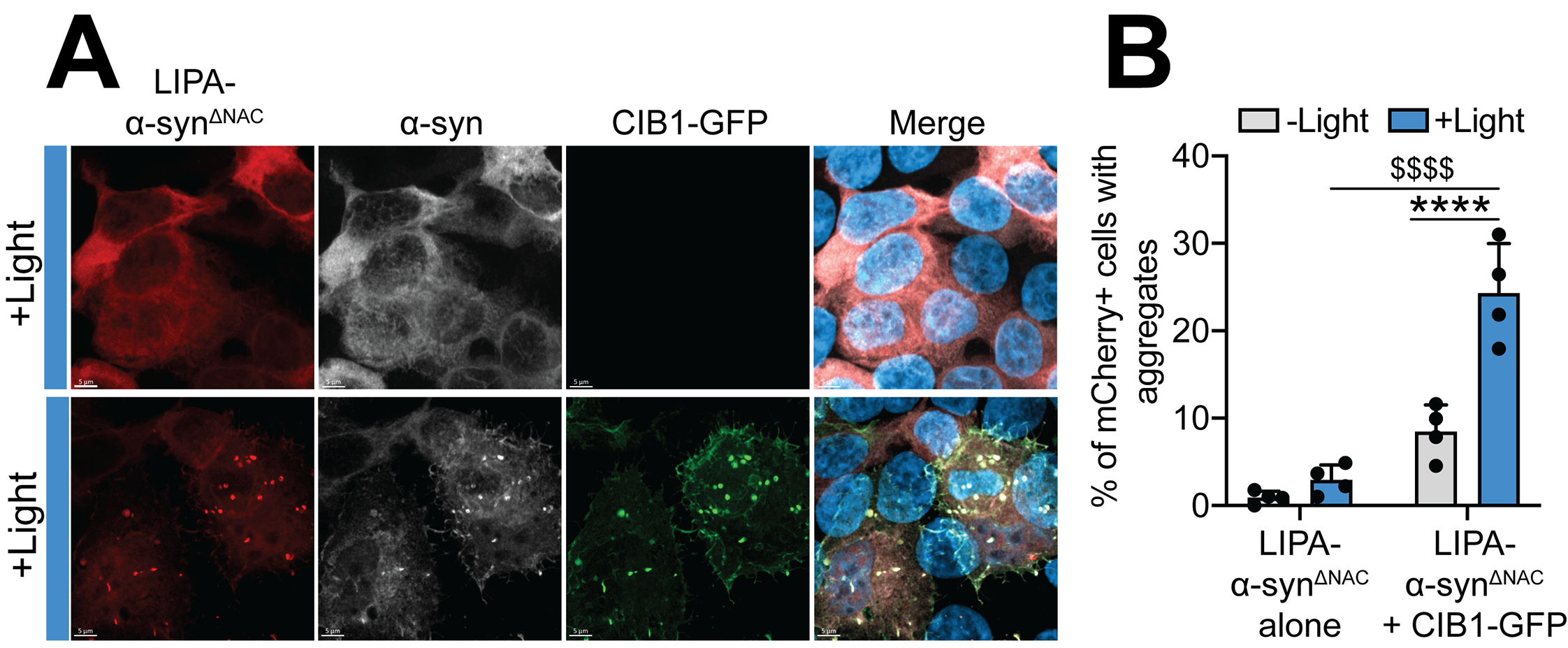

Supplement: S2 Fig — (A) Confocal microscopy images illustrating HEK-293T cells overexpressing LIPA-α-synΔNAC alone or with CIB1-GFP exposed to blue light for 2 hours. Only cells overexpressing LIPA-α-synΔNAC and CIB1 showed mCherry-positive inclusions. These inclusions were also positive for GFP, revealing the coaggregation of the 2 proteins (n = 4) (scale bar = 5 μm). (B) Quantification of mCherry-positive LIPA inclusions, showing a significant increase in the proportion of cells with LIPA aggregates under the condition of both LIPA-α-synΔNAC and CIB1-GFP (n = 4). The data are presented as the means ± SEM. **** p ≤ 0.0001, LIPA-α-synΔNAC + CIB1-GFP + light vs LIPA-α-synΔNAC alone + light and $ $ $ $ p ≤ 0.0001, LIPA-α-synΔNAC + CIBN1-GFP + light vs LIPA-α-synΔNAC + CIB1-GFP–light. The underlying data for (B) can be found in S1 Data. α-syn, α-synuclein; LIPA, light-inducible protein aggregation. (TIF) [file pbio.3001578.s002.tif]

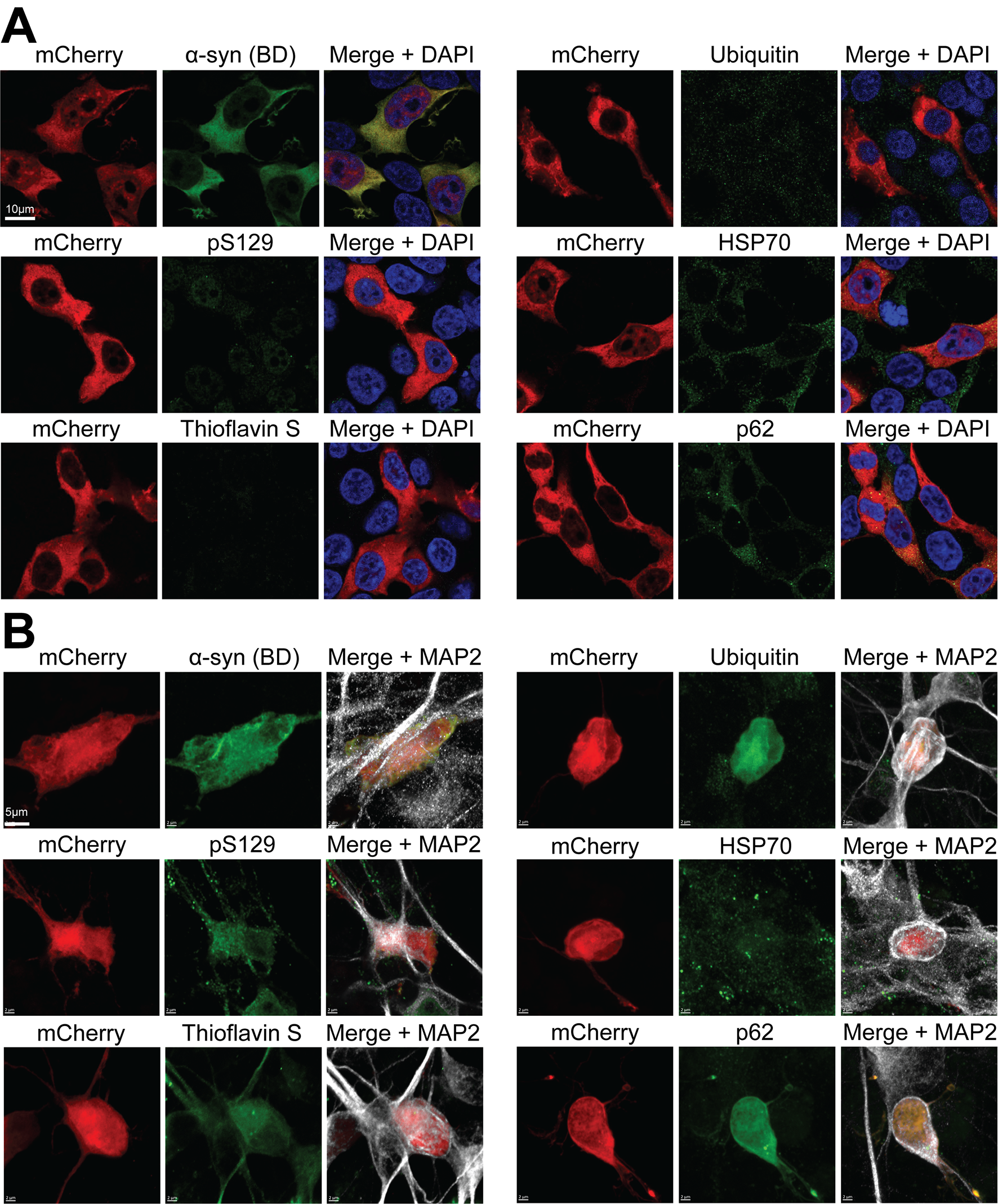

Supplement: S3 Fig — (A) Confocal microscopy images of representative HEK-293T cells and (B) hiPSC-derived neurons overexpressing LIPA-α-syn not exposed to blue light stimulation. Staining with antibodies against α-syn (BDlab), pS129, thioflavin S, ubiquitin, HSP70, and p62 revealed the absence of LB-like inclusions (n = 5) (scale bars = 10 μm and 5 μm in A and B, respectively). α-syn, α-synuclein; hiPSC, human-induced pluripotent stem cell; LB, Lewy bodies; LIPA, light-inducible protein aggregation; pS129, phosphorylated α-syn at S129. (TIF) [file pbio.3001578.s003.tif]

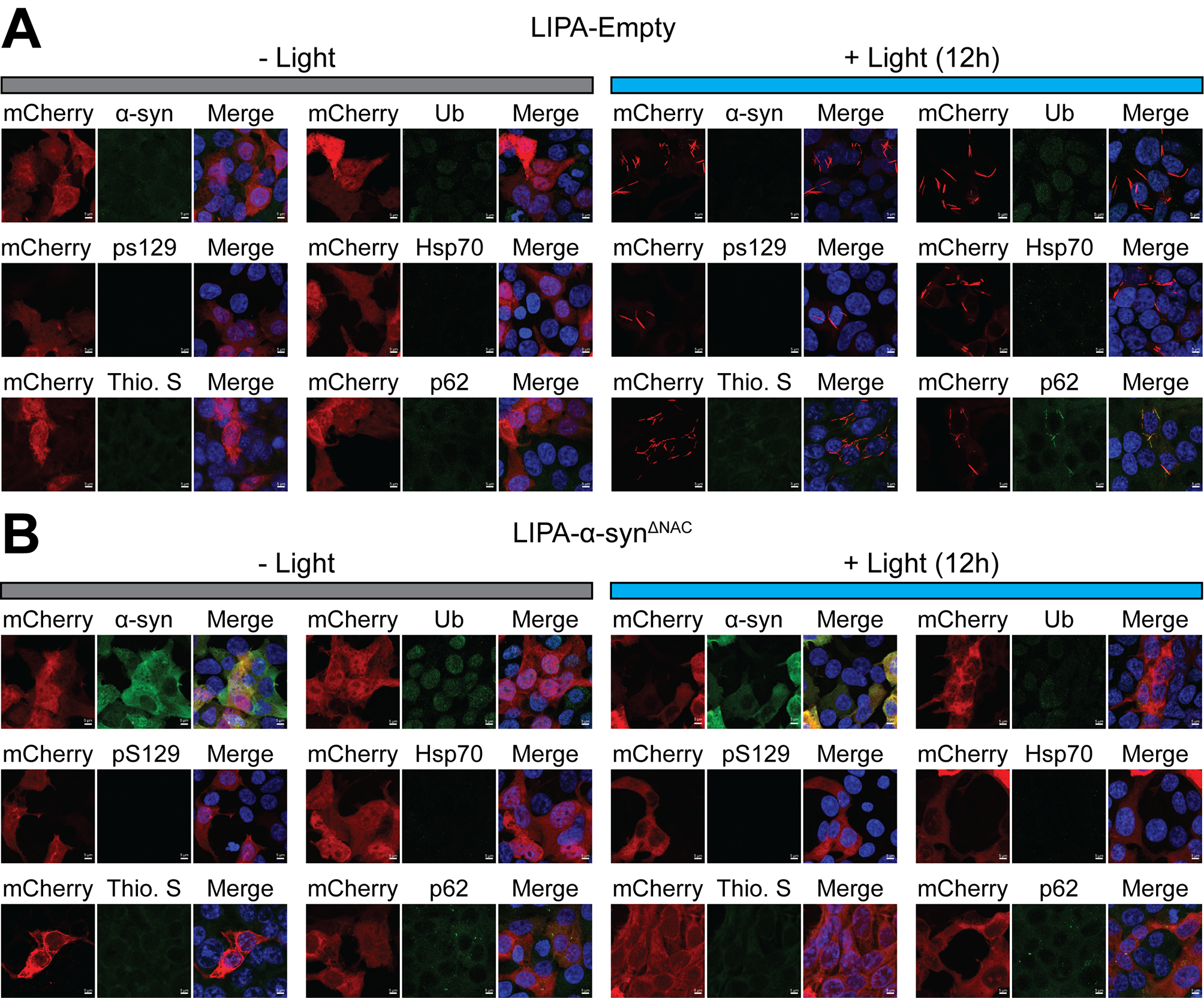

Supplement: S4 Fig — (A) Confocal microscopy images of representative HEK-293T cells overexpressing the LIPA-empty construct (+/−light) and (B) LIPA-α-synΔNAC construct (+/−light). Staining with antibodies against α-syn, pS129, Thio. S, Ub, HSP70, and p62 revealed the absence of LB-like inclusions (n = 3) (scale bars = 5 μm). α-syn, α-synuclein; LB, Lewy bodies; LIPA, light-inducible protein aggregation; pS129, phosphorylated α-syn at S129; Thio. S, thioflavin S; Ub, ubiquitin. (TIF) [file pbio.3001578.s004.tif]

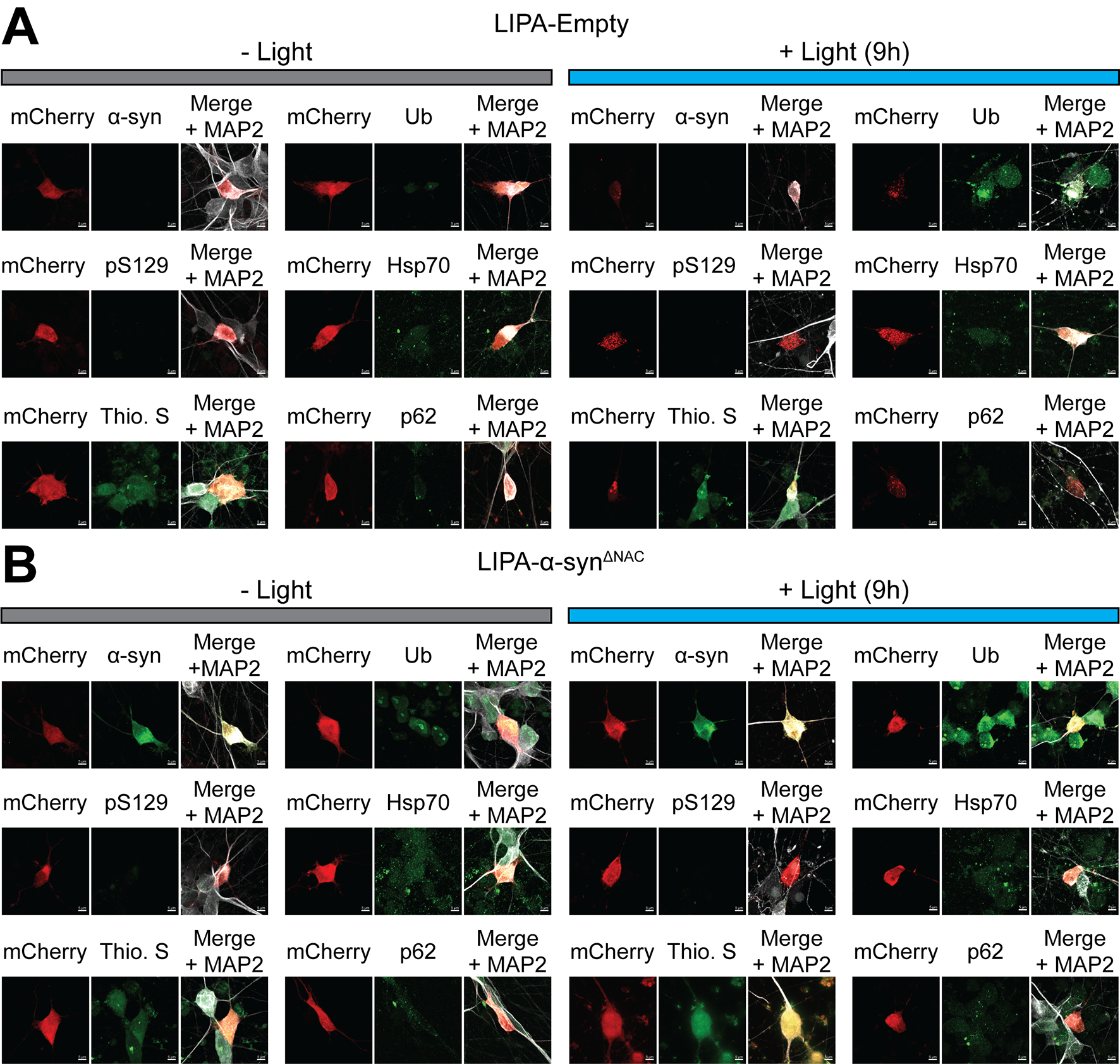

Supplement: S5 Fig — (A) Confocal microscopy images of representative hiPSC-derived neurons overexpressing LIPA-empty construct (+/−light) and (B) LIPA-α-synΔNAC construct (+/−light). Staining with antibodies against α-syn, pS129, Thio. S, Ub, HSP70, and p62 revealed the absence of LB-like inclusions (n = 3) (scale bars = 5 μm). α-syn, α-synuclein; hiPSC, human-induced pluripotent stem cell; LB, Lewy bodies; LIPA, light-inducible protein aggregation; pS129, phosphorylated α-syn at S129; Thio. S, thioflavin S; Ub, ubiquitin. (TIF) [file pbio.3001578.s005.tif]

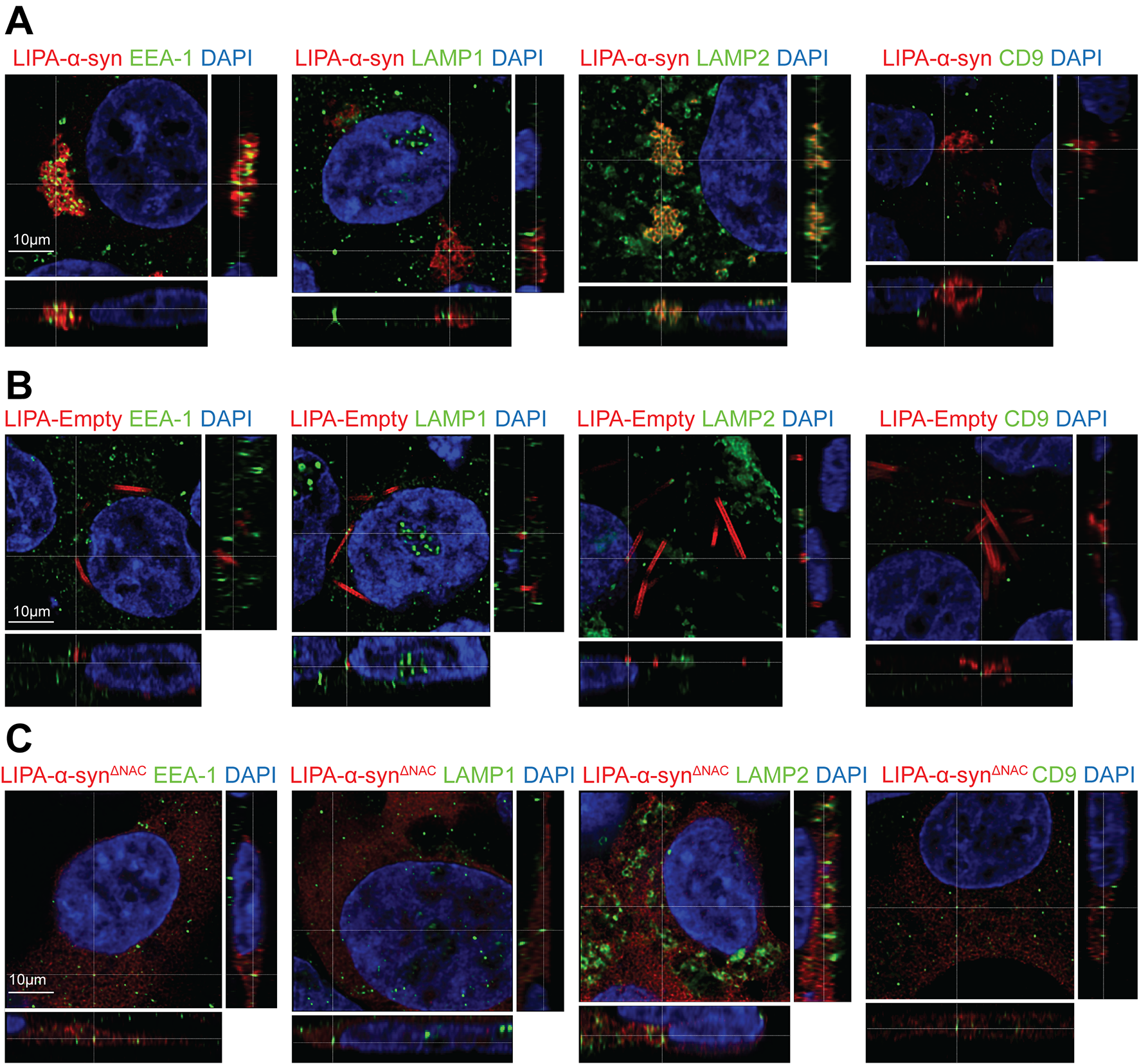

Supplement: S6 Fig — (A) HEK-293T cells overexpressing LIPA-α-syn were exposed to blue light for 12 hours and stained with antibodies against markers of endogenous vesicles. (B) HEK-293T cells overexpressing LIPA-Empty were exposed to blue light for 12 hours and stained with antibodies against markers of endogenous vesicles. (C) HEK-293T cells overexpressing LIPA-α-synΔNAC were exposed to blue light for 12 hours and stained with antibodies against markers of endogenous vesicles. Staining for endogenous vesicles included EEA1, marker of early endosomes; LAMP1 and LAMP2, markers of lysosomes/late endosomes; and staining for CD9, marker of early exosomes (n = 3) (scale bar = 10 μm). α-syn, α-synuclein; LIPA, light-inducible protein aggregation; STED, stimulated emission depletion. (TIF) [file pbio.3001578.s006.tif]

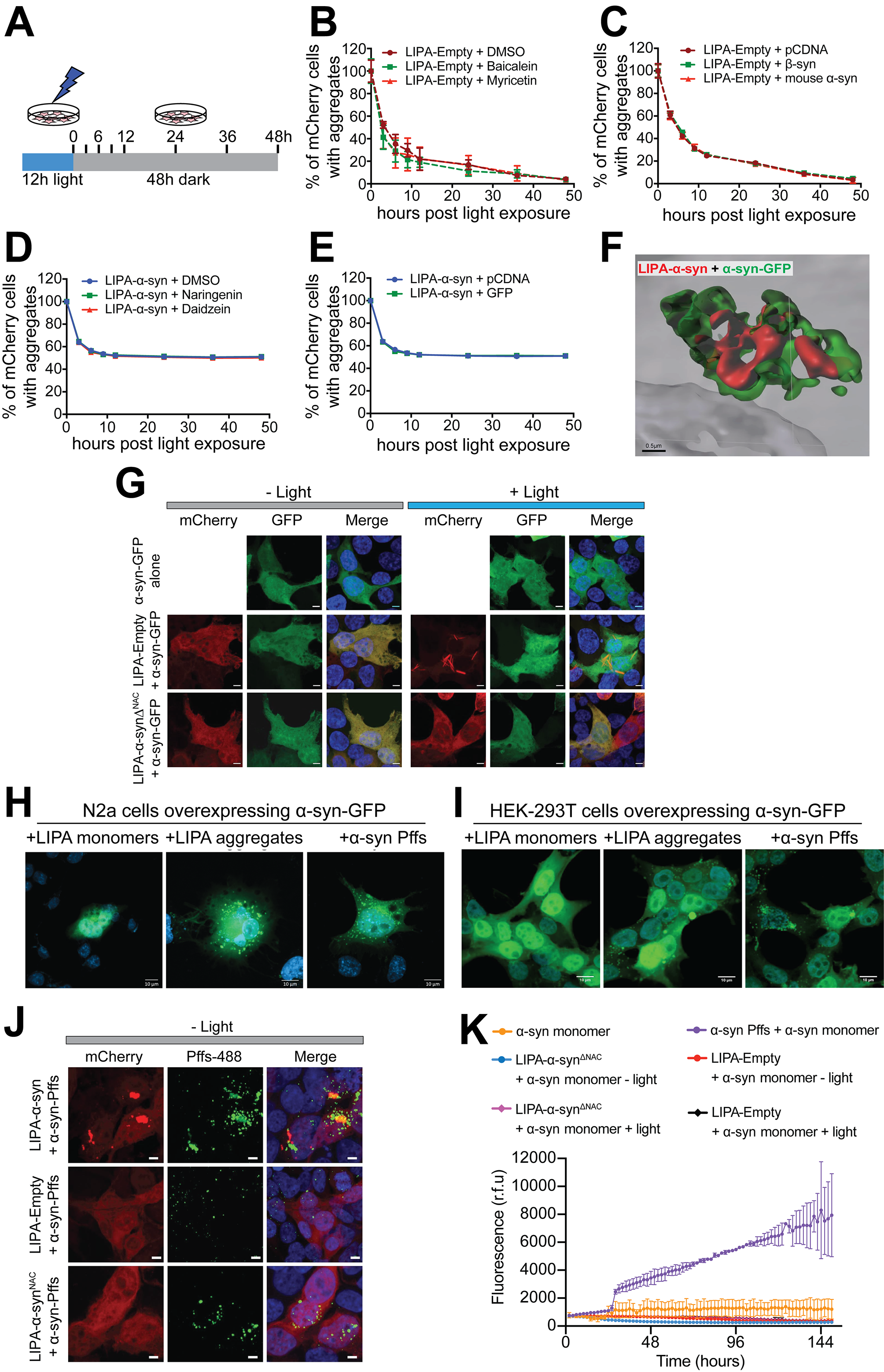

Supplement: S7 Fig — (A) Schematic representation of the experimental paradigm to test LIPA inclusion stability. (B) Time course of LIPA-empty aggregate dissociation in the presence of the α-syn aggregation small-molecule inhibitors baicalein and myricetin (n = 3). (C) Time course of LIPA-Empty aggregate dissociation after the overexpression of β-syn and mα-syn (n = 3). (D) Time course of LIPA-α-syn aggregate dissociation in the presence of small molecules not affecting α-syn aggregation, naringenin or daidzein (n = 3). (E) Time course of LIPA-α-syn aggregate dissociation after the overexpression of GFP (n = 3). The data are presented as the means ± SEM. (F) Three-dimensional reconstitution illustrating the seeding of α-syn-GFP by LIPA-α-syn aggregates as observed after 12 hours of blue light stimulation (scale bars = 0.5 μm). (G) Representative confocal images of HEK-293T cells overexpressing α-syn-GFP alone or the LIPA-Empty or LIPA-α-synΔNAC constructs (scale bar = 5 μm). (H and I) Seeding capacity of LIPA-α-syn aggregates of α-syn-GFP when added to the culture medium with N2a or HEK-293T cells (n = 3), similar observation were collected when α-syn-Pffs were added to the culture medium (scale bars = 10 μm). (J) Representative confocal images of HEK-293T cells overexpressing LIPA constructs and cultured in the presence of α-syn Pff-488 added to the culture medium. Results showed that α-syn Pff-488 were able to seed the aggregation of LIPA-α-syn aggregates in the absence of light stimulation. In contrast, no seeding effect was observed in cells expressing the LIPA-α-synΔNAC or LIPA-Empty constructs (n = 3) (scale bars = 10 μm). (K) RT-QuIC analysis illustrating the kinetics of recombinant α-syn aggregation in the presence of purified LIPA-α-synΔNAC (+/−light), purified LIPA-Empty (+/−light), and recombinant α-syn Pffs. The average ThT fluorescence intensity was plotted against time (n = 3). The data are presented as the means ± SEM. The underlying data for (B), (C), (D), (E), and (K) c [file pbio.3001578.s007.tif]

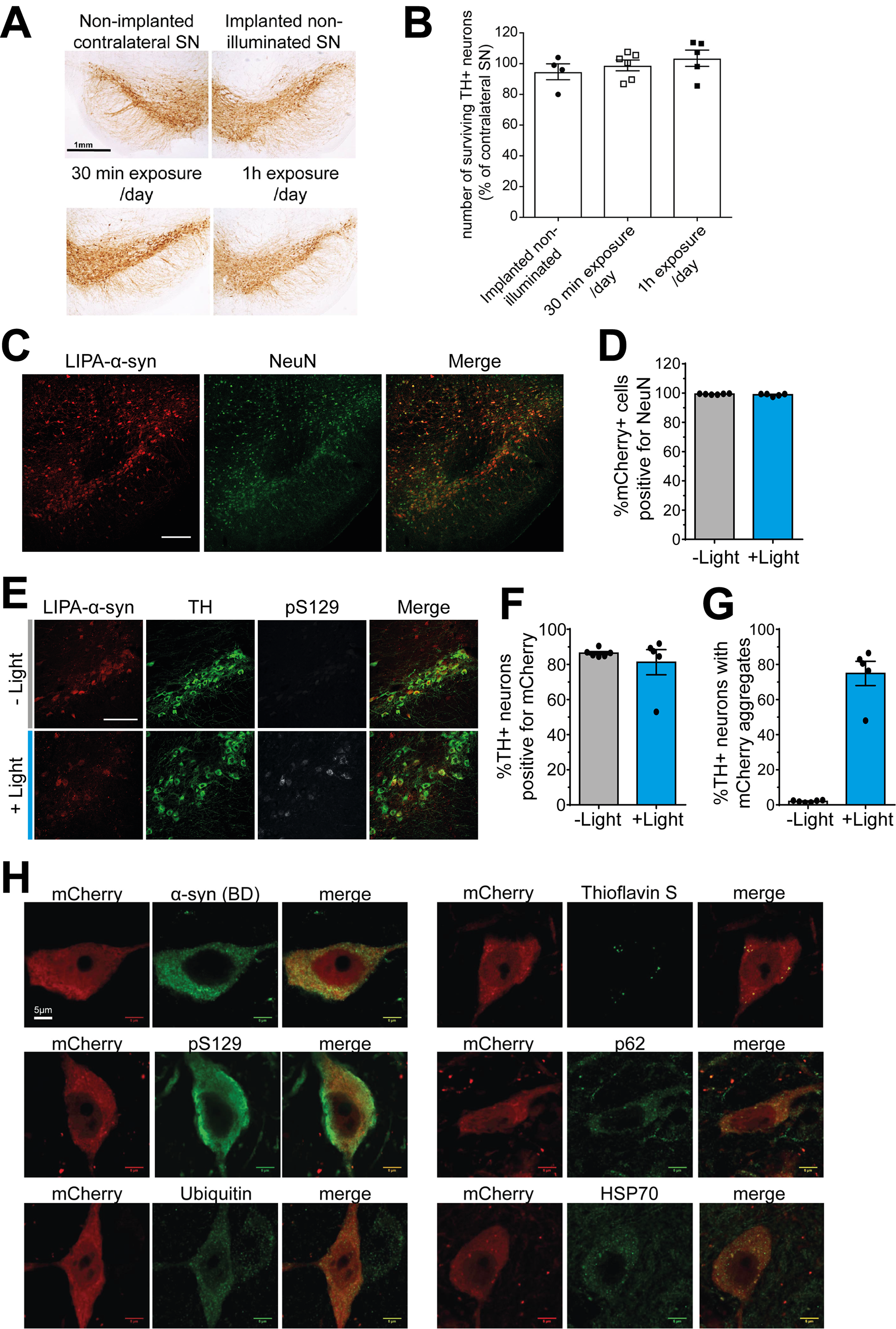

Supplement: S8 Fig — (A) Microscopy images illustrating the density of TH+ dopaminergic neurons in WT mice implanted with optogenetic devices in the midbrain and exposed to blue light for 30 minutes/day or 1 hour/day every other day for 7 days (scale bar = 1 mm). (B) Stereological unbiased quantification of dopaminergic neurons after exposure to blue light for 30 minutes/day or 1 hour/day every other day for 7 days, showing the absence of neuronal loss after light stimulation (n = 4–6 mice). The data are presented as the means ± SEM. (C) Representative confocal images of midbrain neurons (scale bar = 200 μm) and (D) histograms showing the proportion of neurons (NeuN+) overexpressing the LIPA constructs (mCherry+) (n = 5–6 mice). (E) Confocal microscopy images of representative midbrain dopaminergic neurons (scale bar = 200 μm) and (F) histograms showing the proportion of the dopaminergic neurons (TH+) overexpressing the LIPA constructs (mCherry+) (n = 5–6 mice). (G) Histograms showing the proportion of dopaminergic neurons (TH+) depicting LIPA-α-syn inclusions (mCherry+) in the presence or absence of light stimulation (n = 5–6 mice). (H) Confocal microscopy images of representative midbrain neurons not exposed to blue light stimulation showing the absence of LIPA-α-syn aggregates (n = 4 mice) (scale bar = 5 μm). The underlying data for (B), (D), (F), and (G) can be found in S1 Data. α-syn, α-synuclein; LIPA, light-inducible protein aggregation; SN, substantia nigra; WT, wild-type. (TIF) [file pbio.3001578.s008.tif]

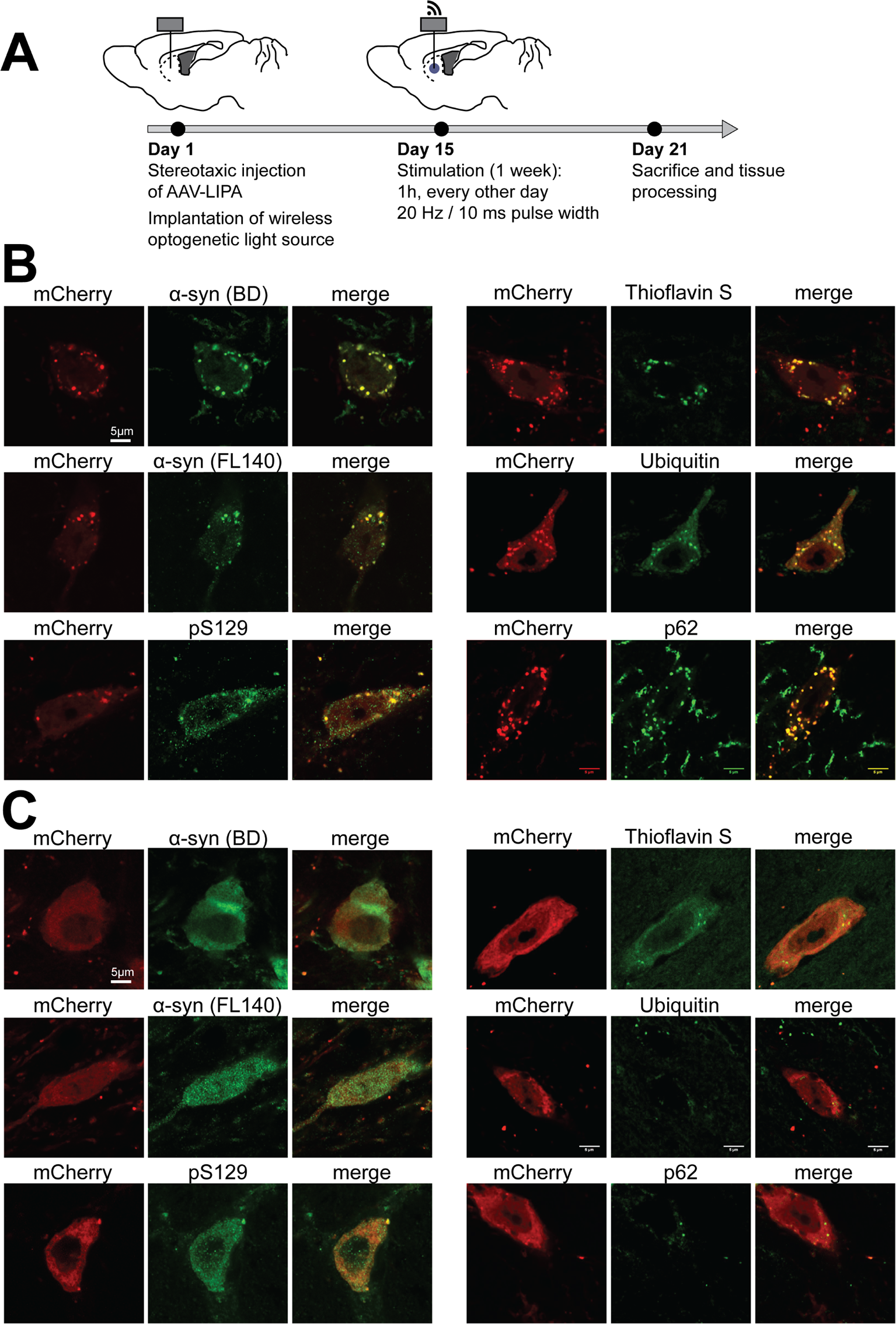

Supplement: S9 Fig — (A) Experimental design of the overexpression and induction of LIPA-α-syn aggregation in the striatum of WT mice. (B) Representative confocal microscopy images of striatal neurons with LIPA-α-syn aggregates exhibiting authentic LB markers: α-syn (BDlab and FL140 antibodies), α-syn pS129, thioflavin S, ubiquitin, and p62 (n = 5 mice) (scale bar = 5 μm). (C) Confocal microscopy images of representative striatal neurons overexpressing LIPA-α-syn not exposed to blue light stimulation and stained with authentic LB markers: α-syn (BDlab), pS129, thioflavin S, ubiquitin, HSP70, and p62 (n = 5 mice) (scale bar = 5 μm). AAV, adeno-associated virus; α-syn, α-synuclein; LB, Lewy bodies; LIPA, light-inducible protein aggregation; pS129, phosphorylated α-syn at S129; WT, wild-type. (TIF) [file pbio.3001578.s009.tif]

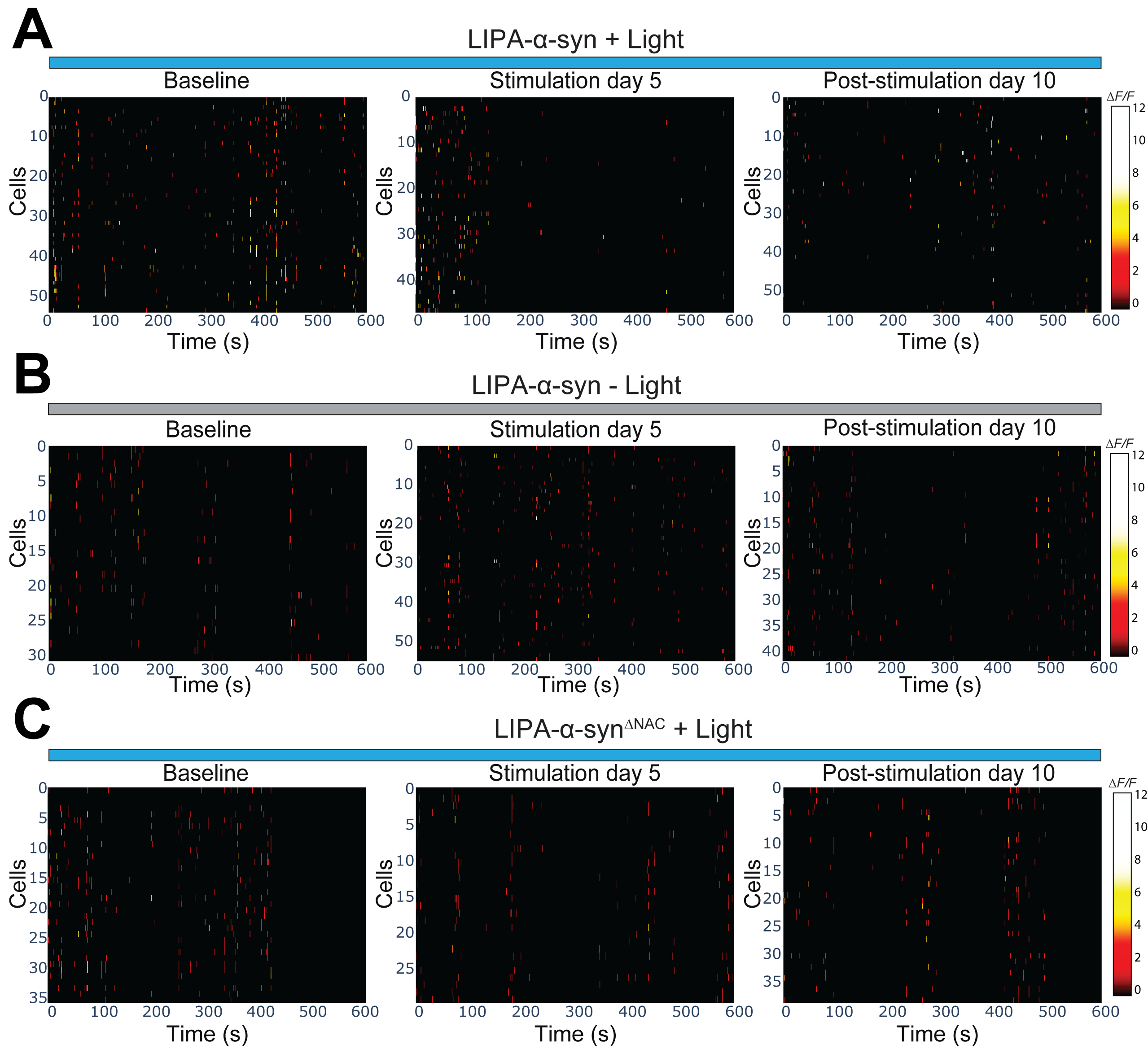

Supplement: S10 Fig — Representative heat maps of Ca2+ signals in one animal overexpressing (A) LIPA-α-syn exposed to blue light, (B) LIPA-α-syn without light stimulation, and (C) LIPA-α-synΔNAC exposed to blue light. Analysis was performed before (Baseline), during (Stimulation day 5), and after (Poststimulation day 10) optogenetic stimulation. The underlying data for (A), (B), and (C) can be found in S1 Data. α-syn, α-synuclein; LIPA, light-inducible protein aggregation. (TIF) [file pbio.3001578.s010.tif]
