# Supplementary material for: A light-inducible protein clustering system for in vivo analysis of α-synuclein aggregation in Parkinson disease
Source: PLoS Biol. 2022 Mar 9;20(3):e3001578. doi: 10.1371/journal.pbio.3001578 (PMC8936469; doi:10.1371/journal.pbio.3001578)
Supplement: S1 Raw images — (PDF) [file pbio.3001578.s018.pdf]

S1_raw_images

Figure 1D

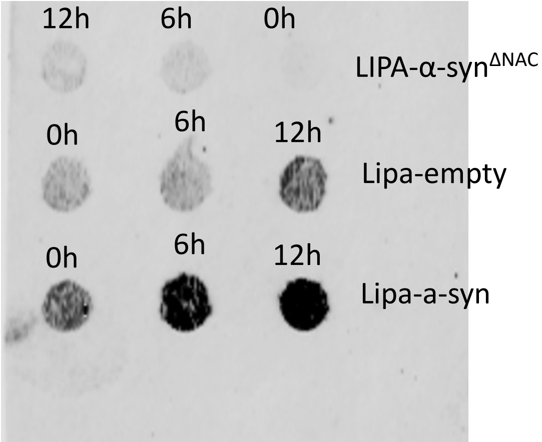

Figure 1E

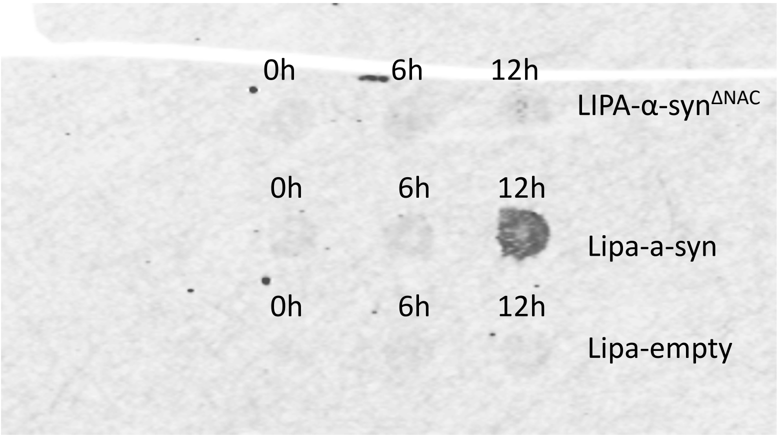

Figure 1F

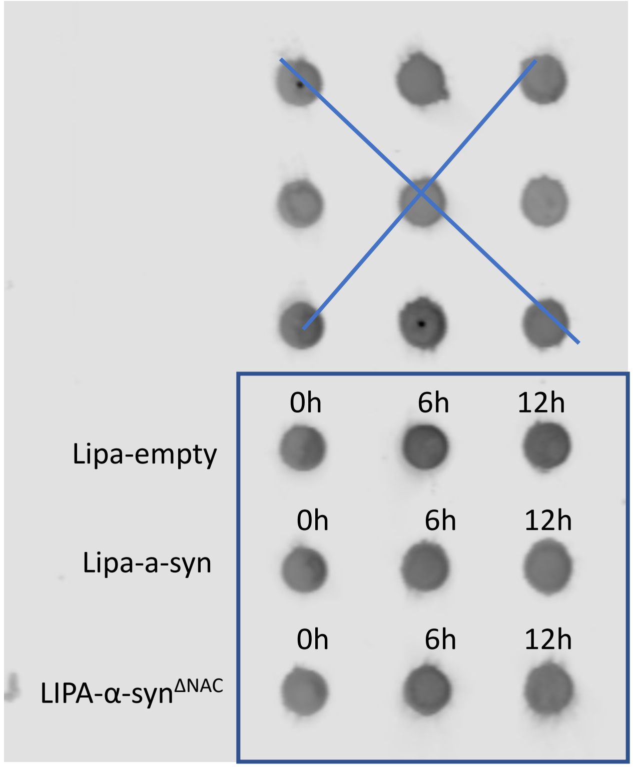

Visualization and quantification were carried out with the LI-COR Odyssey scanner and software (LI-COR Lincoln, NE, USA).

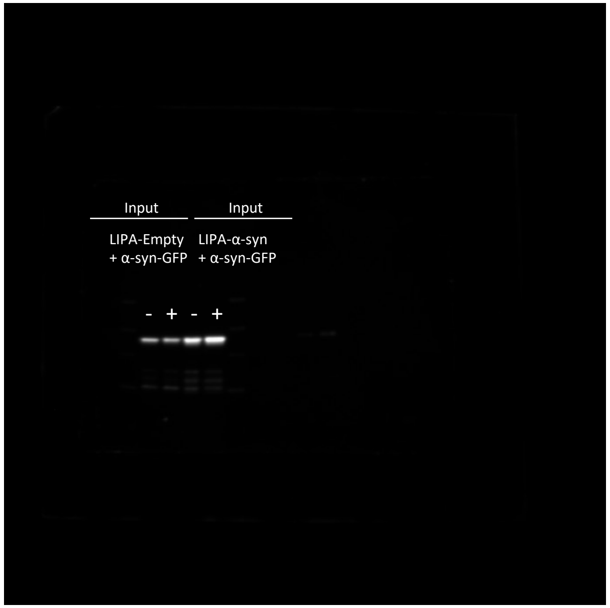

Original

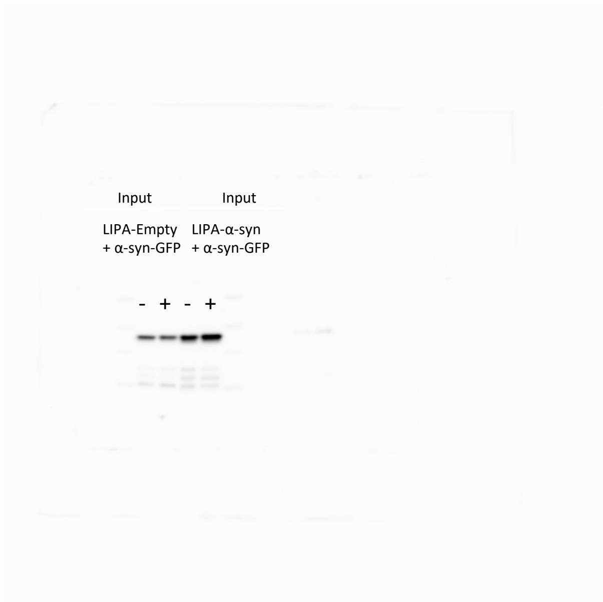

Inverted

Figure 3E
GFP-HRP panel
Low exposure
Used in Figure 3E input

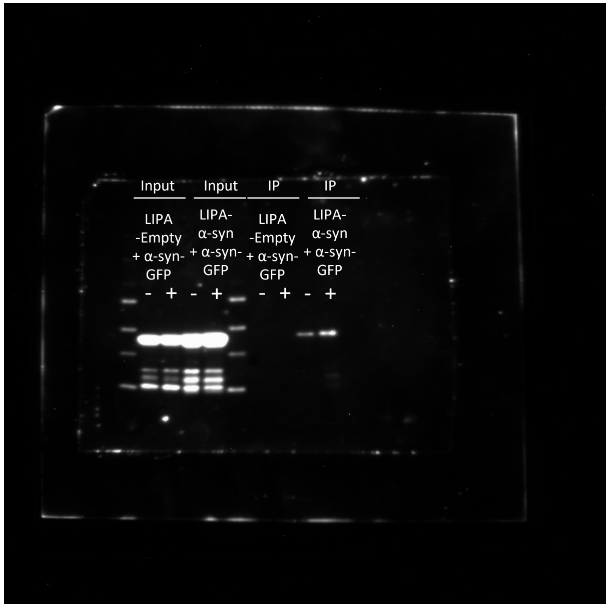

Original

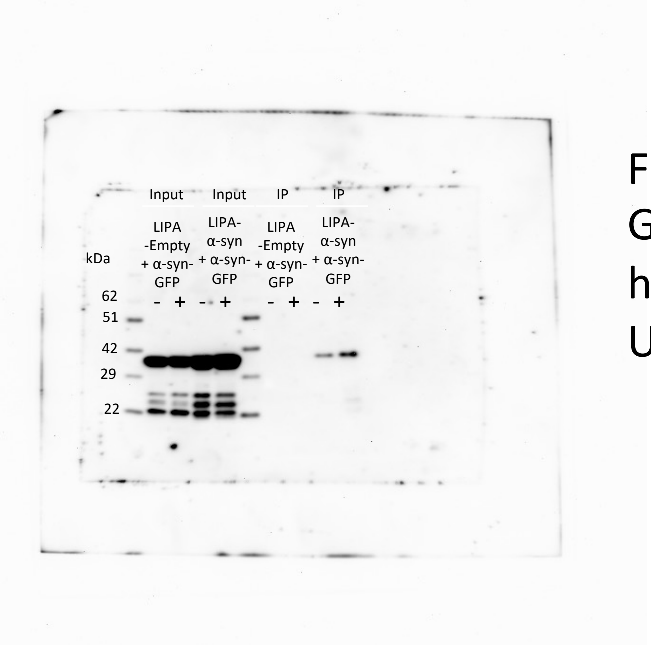

Inverted

Figure 3E
GFP-HRP panel
high exposure
Used in Figure 3E IP

The blots were imaged using the Thermo Scientific™MYECL™ Imager

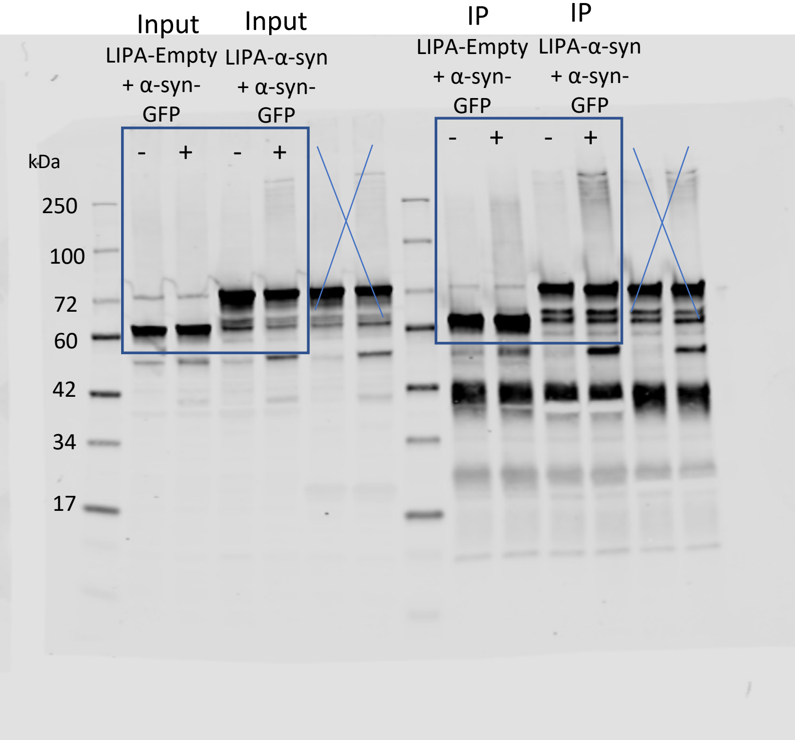

Figure 3E
Mcherry aggregates and mcherry panel

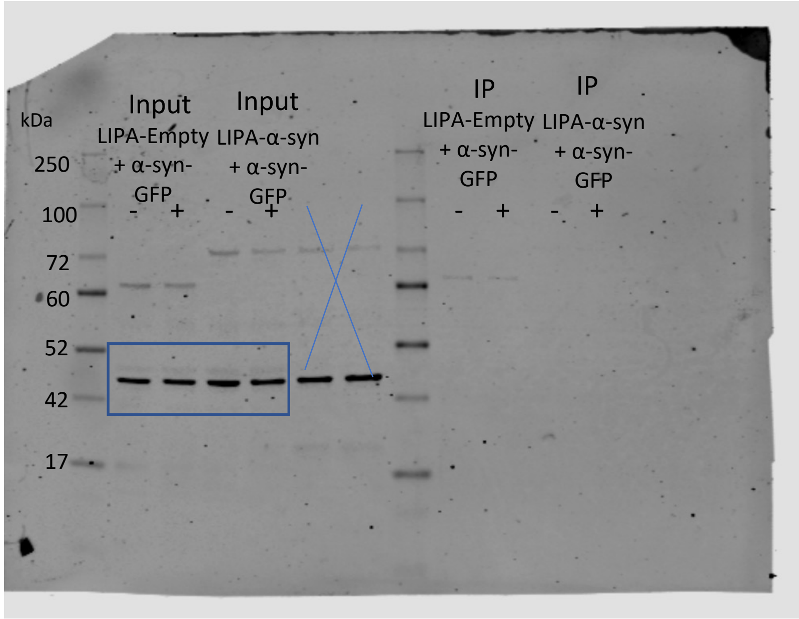

Figure 3E
Actin panel

Visualization was carried out with the LI-COR Odyssey scanner and software (LI-COR Lincoln, NE, USA).

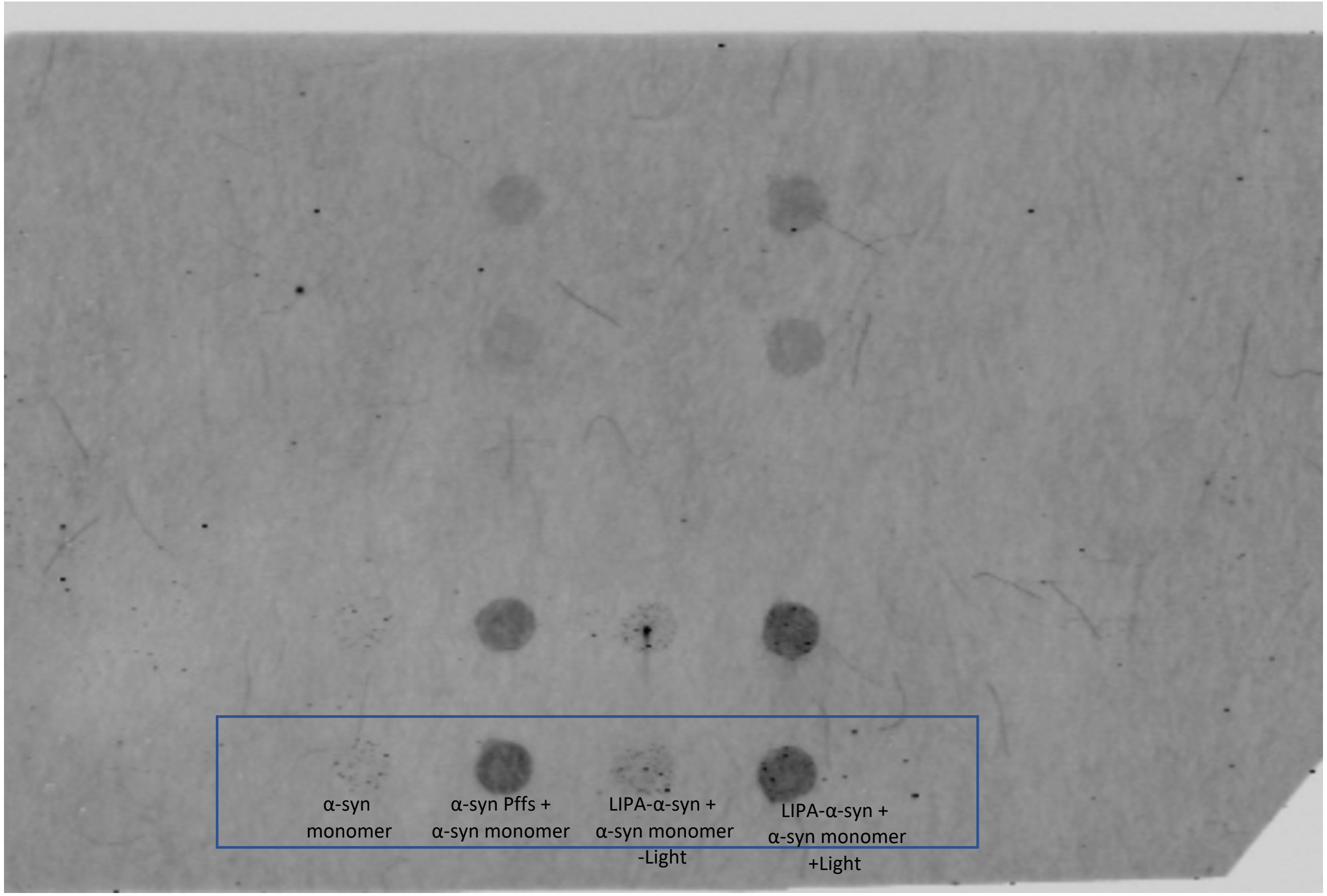

Figure 3J
Filter retardation blot

Visualization and quantification were carried out with the LI-COR Odyssey scanner and software (LI-COR Lincoln, NE, USA).

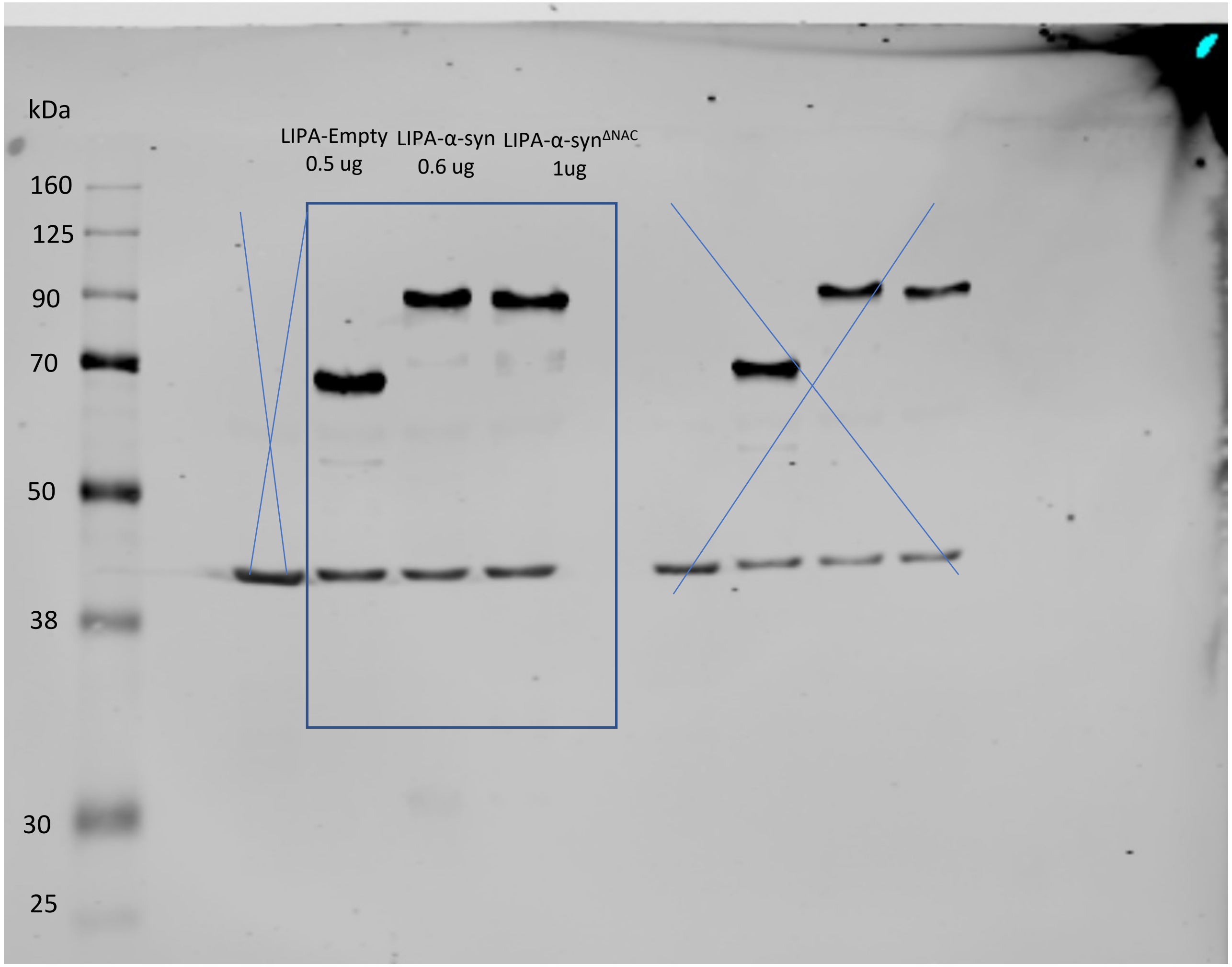

Figure S1A

Visualization and quantification were carried out with the LI-COR Odyssey scanner and software (LI-COR Lincoln, NE, USA).
