# Supplementary material for: A light-inducible protein clustering system for in vivo analysis of α-synuclein aggregation in Parkinson disease
Source: PLoS Biol. 2022 Mar 9;20(3):e3001578. doi: 10.1371/journal.pbio.3001578 (PMC8936469; doi:10.1371/journal.pbio.3001578)
Supplement: Attachment — Submitted filename: Response to Reviewers .pdf [file pbio.3001578.s019.pdf]

UNIVERSITÉ LAVAL
Faculté de médicine
Département de médicine moléculaire

CHU de Québec
Centre de Recherche
Axe des Neurosciences

Response to Reviewers

Reviewer # 1

1- Authors should site the original work that developed optoDroplet method (PMID: 28041848). In this regard, the last sentence of Abstract "Collectively, our findings provide a unique tool for the generation, visualization, and dissection of the role of protein aggregation in neurodegeneration" is overstated and need to be more specific. Also, the title needs to be more specific, too, as the basic principle has been published

Re: As per the reviewer's suggestion, we have added the citation of the original work reporting on the development of the optodroplet method (reference # 13). We have also modified the title and the abstract to better reflect the nature of our work on α-synuclein accumulation in Parkinson's disease.

2- CRY2-based optogenetic method can temporally control protein-protein interaction, but it is still difficult to identify which intermediate species that form during the assembly of oligomer and aggregates are cytotoxic. As mentioned in the discussion, it is still possible that the LIPA-α-syn aggregates are the product of neuroprotective response of cells. The authors need to weaken the claim like, "In the present study, we reported that α-syn aggregation induced a significant loss of DA neurons in the midbrain that was correlated with motor dysfunction." (p17). Also, regarding the first sentence of Abstract "Neurodegenerative disorders refer to a group of diseases triggered by the aggregation of normal proteins into proteinaceous inclusions.", it is not known whether neurodegeneration is "triggered" by protein aggregation

Re: As per the reviewer's suggestion, we made the necessary changes to the abstract and discussion (page 18).

3- It seems that LIPA-α-syn aggregation (LLPS?) first takes place in the nucleus immediately after light illumination and cytoplasmic aggregation comes after (Sup Video2). The nuclear aggregation/LLPS is also detectable in LIPA-α-synΔNAC, but cytoplasmic aggregation did not follow (Sup Video3). The nuclear aggregation appears less evident in LIPA-Empty (Sup Video1). Is it possible that LIPA-α-syn phase transition occurs in a step-wise manner in different subcellular compartments? It would be interesting to know what the subnuclear structure of LIPA-α-syn that appears right after the illumination is. The authors at least mention to this LIPA-α-syn-positive subnuclear structure, even if they think these are not important.

Re: We thank the reviewer for pointing out this relevant observation. We have now described the possible differential LIPA aggregation in various subcellular compartments, including the nucleus, and discussed this in the main text (pages 17 and 18).

Reviewer # 2

1- Although all the necessary controls are included, I wonder why the light control is not included. Can a-Syn-(WT) mCherry alone induce aggregate upon blue light exposure?

Re: To rule out the possibility that the mCherry tag could promote the aggregation process of the LIPA constructs per se, we have added data showing that HEK-293T cells overexpressing α-syn-mCherry (without CRY2olig), exposed or not exposed to the blue light, do not display mCherry-positive inclusions (page 5 and Figure S1D).

2- In Figure 1F, please described the loading control how it is determined (I guess nitrocellulose retained all of the protein).

Re: We have taken 20-30% of the total sample volume before the addition of SDS and exposed these samples to PBS-soaked nitrocellulose membranes (0.2 µm). By using nitrocellulose, we ensured that it retained all proteins. All subsequent experimental procedures associated with the positive control condition were performed in PBS without SDS. This has now been described in the Materials and Methods section (page 31) and the choice of nitrocellulose mentioned in the figure legend.

3- Figure 2C-H. LIPA-a-Syn inclusions using TEM are not clear. Where are the aggregates? The legends (arrow etc.,) appear to show the organelles. The LIPA-a-Syn aggregates presence Figure 2C-H are unclear. The author may have to include TEM images of LIPA-a-Syn mutant control to distinguish the aggregates.

Re: As per the reviewer's suggestion, we have added representative TEM images of control cells to showcase the ultrastructure of the LIPA-α-syn inclusions. We have added a representative TEM image of a cell overexpressing LIPA-α-syn without aggregates (- Light) and a cell harboring LIPA-Empty inclusion (Figure 2C and E).

4- Figure S4, STEM images should also include LIPA-empty or LIPA-a-Syn to appreciate the light-induced aggregate distribution.

Re: We have added STED images of LIPA-Empty and LIPA-α-syn$^{\Delta NAC}$ (Figure S6).

5- Figure 3. It is unclear whether a-Syn-GFP forms aggregates due to light and how does a-Syn-GFP alone (without LIPA-a-Syn) look when exposed to blue light. Alternatively, LIPA-a-Syn mutant control in the seeding experiments in Figure 3D would be beneficial.

Re: We have confirmed that α-syn-GFP overexpression alone, exposed or not exposed to the blue light, does not form aggregates. We have also added controls by overexpressing α-syn-GFP with LIPA-Empty and LIPA-α-syn$^{\Delta NAC}$ and did not observe protein seeding of α-syn-GFP in these conditions (main text page 11 and Figure S7G).

6- It is related to the above question, what percentage of LIPA-a-Syn turned into aggregates upon blue light exposure? Data show (Fig 2 B, D) that the blue light effect is time-dependent, but it is not clear that blue light ultimately converts all the soluble a-Syn into aggregated form.

Re: Using live imaging, we performed a quantification of the LIPA-α-syn intensity in the cytosol and measured that 55-60% of the LIPA-α-syn monomers are converted to aggregates after 180 min of light stimulation (please see Figure below).

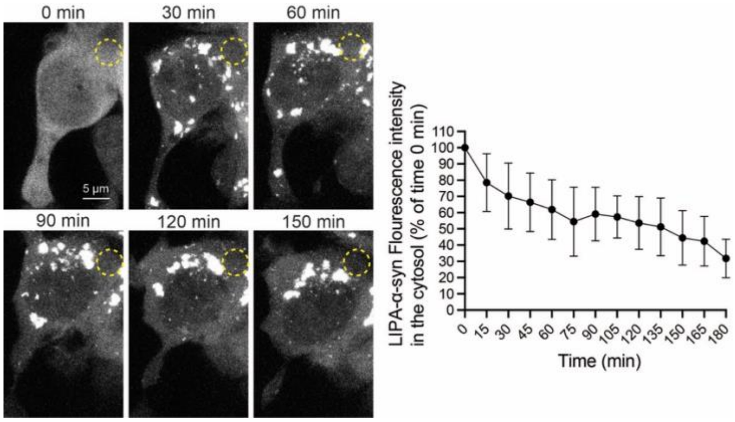

Figure 1: Estimation of LIPA-α-syn monomer conversion to inclusions following light stimulation. Time-lapse live imaging illustration of representative HEK-293T cells overexpressing the LIPA-α-syn and exposed to the blue light (scale bar=5 µm). The dashed yellow line delineates the region of interest where analysis of the mCherry fluorescence intensity was performed. Estimation of the relative mCherry intensity in the region of interest at different time points of light stimulation (n=17 cells).

7- First paragraph "Survival of striatal neurons." I guess this should be mid-brain DA neurons.

Re: We thank the reviewer for pointing out this error. The modification has been made to the revised manuscript (page 15).

8- LIPA-induced α-syn aggregates precipitate dopaminergic neuronal loss and induce parkinsonian- like symptoms. Under this section, authors discuss Lewy body (LB)'s role as protective vs. toxic and proclaim their data indicates LB-like LIPA-a-Syn aggregates are toxic. LIPA-a-Syn in their system may exist as LB as well as oligomeric species, which are considered harmful. Authors own data, western blot Figure 3 indicates besides higher-order (non-gel penetrating, top of the gel) LIPA-a-Syn species, there is also oligomeric (gel-entered, middle of the gel) LIPA-a-Syn species. So, soluble and oligomeric species of LIPA-a-Syn might be causing toxicity, not the LB-like aggregate species. Contemplating this possibility in the discussion may be helpful.

Re: As per the reviewer’s comment, we have modified the text and discussed the potential role of soluble and oligomeric forms of α-syn to neuronal toxicity (pages 18 and 19).

Reviewer # 3

1- Fig. 1: Without details on the expression levels of all transgenes, this figure is inconclusive. The linearity of the assay is not obvious and a negative control is missing. SDS-PAGE and anti-mCherry blot would be more convincing because the kDa resolution would also allow the reader to assess degradation problems, SDS-stable aggregates etc. Fig. 3F, 3rd lane, suggests aSyn-LIPA may express higher than empty-LIPA. If that is the case, the experiment is inconclusive.

Re: To ensure equal protein expression levels for all the LIPA constructs, we performed transient transfection with different amount of DNA for each plasmid coding for each construct in HEK-239T cells and we evaluated the mCherry protein levels by Western blot. Using this approach, we were able to determine the amount of DNA for each construct to be used in order to obtain equal protein expression (0.5 µg of LIPA-Empty, 0.6 µg LIPA-α-syn and 1 µg LIPA-α-syn$^{\Delta NAC}$ per well in a 6-well plate). This information has now been added to the main text (page 5), Figure S1A as well as to the Materials and Methods section (page 24).

2- Fig. 2: This figure would be a lot stronger with more controls. Cells containing LIPA-empty inclusions as shown in Fig. 1 would be very good controls. The more transient, presumably not beta-sheet-rich, LIPA-empty inclusions should stain negative for ThioS and not overlap with the other markers as well. Using this control in a consequent manner would also dissipate any doubt about the true nature of the signals (bleed-through from other channels etc.) The authors are strongly encouraged to add this control. It would also be good to see what LIPA-empty inclusions look like by EM.

Re: As per the reviewer's suggestion, we have added control conditions (LIPA-Empty and LIPA-α-syn$^{\Delta NAC}$) and confirmed the absence of LB-like marker accumulation in these conditions, in HEK-239T-cells and in hiPSC-derived neurons (Figure S4 and S5). We have also added a representative TEM image of a cell overexpressing LIPA-Empty and exposed to the blue light (Figure 2E).

3- There also seems to be some disconnect between the apparent lack of fibrils by EM and the ThioS staining. Wouldn't one expect that sth that stains clearly for ThioS is largely fibrillar in the cell? If not: are smaller aggregates sufficient for ThioS staining? What is the nature of the aggregates is definitely a key question here... The SDS-stable aggregates in Fig. 3E are low in abundance. The fibrillar nature of the purified material is not beyond any doubt since it could be a post-lysis artefact. Shahmoradian et al actually suggested that LBs are largely devoid of fibrillar material, and your data are in line with that, which is actually quite striking. The ThioS data, however, may be in contrast to this. In my own experience, and talking to colleagues over the years, using ThioS staining on cells is tricky and needs very good controls to be convincing... ThioS may also bind to vesicle aggregates due to its hydrophobic nature.

Re: We agree with the reviewer's comment that Thioflavin S staining is tricky and require the use of controls. In our study, the use of negative controls (LIPA-α-syn -blue light or in LIPA-Empty and LIPA-α-syn$^{\Delta NAC}$ +/- blue light) did not show any Thiofalvin S staining. In contrast, LIPA-α-syn aggregates (+ blue light) showed such staining. Moreover, we confirmed these observations using another fluorescent dye that binds specifically to the β-sheet structure of amyloid-like protein aggregates, amytracker (PMID: 29445039, 32075919) (please see figure below).

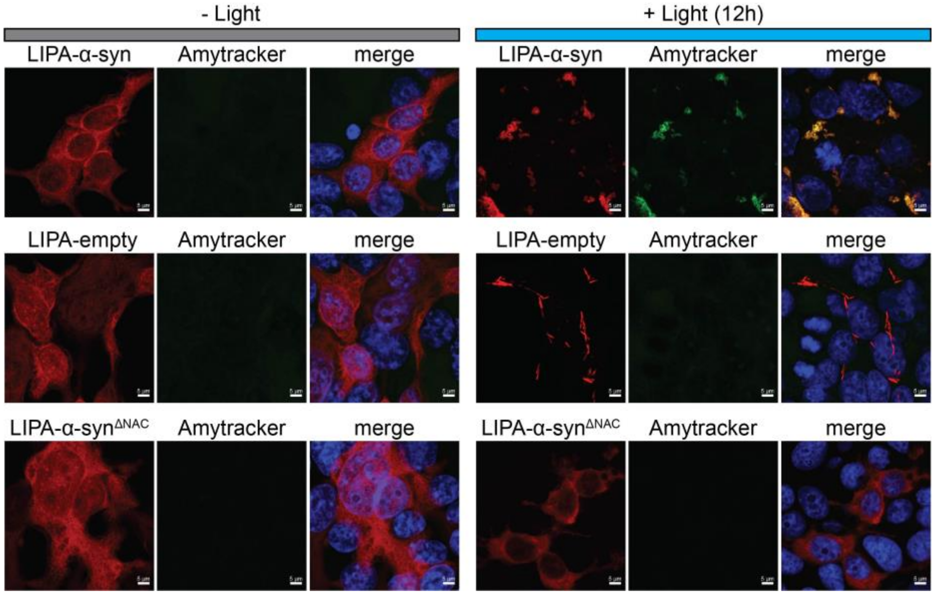

Figure 2: Amytracker® specifically stains LIPA-α-syn. Representative confocal microscopy images of HEK-293T cells overexpressing the LIPA-α-syn construct (-/+ light, top lane), LIPA-empty construct (-/+light, middle lane) or LIPA-α-syn$^{\Delta NAC}$ construct (-/+ light, bottom lane) and stained with the Amytracker®, a luminescent conjugated oligothiophene. Results revealed an accumulation of β-sheet structures in the LIPA-α-syn condition while the signal was absent in the LIPA-empty and LIPA-α-syn$^{\Delta NAC}$ condition (n=3) (scale bars= 5 µm).

4- Fig. 3: It would be great to see the results of the following experiment: Cells expressing LIPA-empty or LIPA-aSyn plus minus PFF, plus minus light. PFFs should seed LIPA-aSyn just fine even without light. LIPA-empty should be unaffected. It'd be very interesting to see the effect of light. Fig. 3D is very impressive. It is also striking that in Fig. 3 aggregated aS was indeed taken up.

Re: As per the reviewer's suggestion, we performed these experiments and as he/she anticipated, in the absence of light, α-syn Pffs are able to specifically seed the aggregation of LIPA-α-syn, but not the LIPA-Empty nor LIPA-α-syn$^{\Delta NAC}$. This data has now been added in the main text (page 11) and in Figure S7J.

5- Fig. 4/5: These are quite impressive, complicated experiments. Yet, controls would make the data more conclusive. What is shown - please correct me if I am wrong - is consistent with the simple notion that expressing a stressor in a certain brain area causes problems in that brain area. Expressing a control fusion (TDP43-LIPA?) in the same regions leading to different results would help establish aSyn-specificity and PD relevance better.

Re: We thank the reviewer for his/her suggestion. We agree that expressing a control fusion protein (i.e. TDP-43) may indeed generate more confidence regarding the specific toxicity of LIPA-α-syn aggregates. However, these experiments may take several months and we think that this question can be addressed in follow up study. In addition, we believe that the absence of neuronal toxicity after LIPA-Empty aggregation or after LIPA-α-syn$^{\Delta NAC}$ overexpression using our experimental conditions supports the specific toxicity of LIPA-α-syn aggregates.

6- It would also be good to know if the transgenes are sorted correctly to synapses, and what happens to the endogenous protein when the transgene is there and caused to aggregate.

Re: Using our experimental conditions, we were able to observe that the LIPA-α-syn was correctly sorted to the synapse and co-localizes with the presynaptic maker, synaptophysin, in the dopaminergic terminals in the striatum (please see figure below). This concept of α-syn aggregation transport to the synapses and its impact on the synaptic activity is currently being investigated in an independent project.
Regarding the interaction of LIPA-α-syn aggregates with the endogenous mouse α-syn, the absence of commercially available antibodies allowing to discriminate between the human and the mouse α-syn unfortunately precluded this analysis.

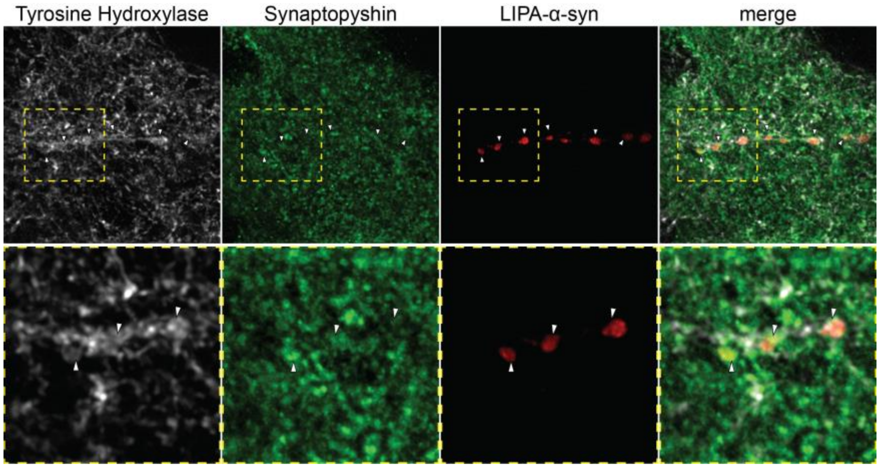

Figure 3: STED imaging reveals the presence of LIPA-α-syn in the dopaminergic synaptic terminal in the striatum. Representative STED images of dopaminergic fibers in the striatum (white, tyrosine hydroxylase) exhibiting LIPA-α-syn expression (red, arrowheads) at the synaptic terminals (synaptophysin, arrowheads). The lower panel represents higher magnification images of the regions surrounded by the yellow square (upper panel).

7- Mouse aSyn slows down human aS aggregation, but would mouse aSyn still be seeded to aggregate?

Re: The question of the possible seeding of LIPA-syn and mouse α-syn is very relevant. Unfortunately, the commercially available antibodies recognize both human and mouse α-syn precluding the discrimination between the protein of the two species in the protein inclusion.

8- Would human-aSyn-LIPA induced aggregates spread to other neurons to cause mouse aSyn to aggregate? Does LOF of endogenous mouse aSyn play any role here, or is it all GOF of human LIPA-aSyn?

Re: Under our experimental condition, we were indeed able to observe cell-to-cell propagation of LIPA-α-syn aggregates in cell culture. However, we feel this observation is beyond the scoop of the current manuscript and is therefore being investigated as part as a distinct project.

9- The presence of the large mCherry tag could be addressed better. This may affect aSyn fibrillization etc. The method doesn't really need the tag, unless for visualization. Have you considered to do a subset of expts without the tag?

Re: In the current study we used tagged LIPA-α-syn. However, we have confirmed that LIPA-α-syn with or without the mCherry tag are able to aggregate and exhibit similar LB-like biochemical characteristics (see Figure below). In future studies, we are considering using untagged constructs.

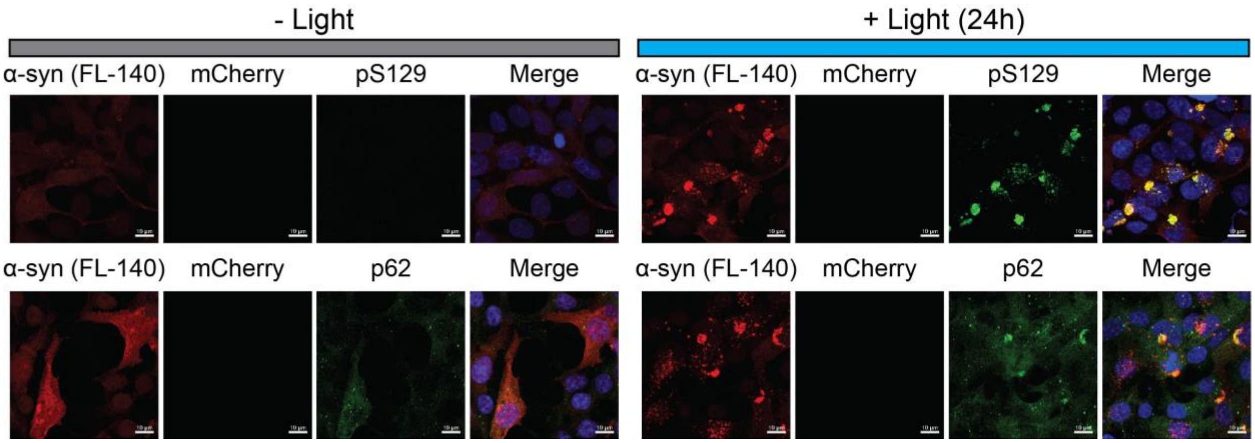

Figure 4: mCherry tag is not required for Cry2-mediated α-syn aggregation. Representative confocal microscopy images of HEK-293T cells overexpressing the α-syn-CRY2olig construct without the mCherry tag (-/+ light). Cells were co-stained for α-syn (red, FL-140 clone) and phosphorylated α-syn at S129 (pS129, green, top lane) or p62 (green, bottom lane). Cells were exposed to blue light for 12 hours and exhibited α-syn aggregates independent of the mCherry tag (n=3) (scale bars= 10 µm).

10- Not least due to the tag, plus the forced dimers as subunits, statements about "authentic LB formation" may have to be toned down. The nature of LBs is under debate, and it is not clear if the inclusions here are closely similar to aSyn in LBs.

Re: As per the reviewer's suggestion, we toned down the statement regarding the resemblance between the LIPA-α-syn aggregates and authentic LBs.

11- Carrying deltaNAC and empty controls more through the paper, plus adding a control of another aggregation-prone protein to establish aSyn/PD specificity could have added more clarity.

Re: We have added control conditions (LIPA-Empty and LIPA-α-syn$^{\Delta NAC}$) to further support our conclusions (Figures S4, S5, S6, S7G and J). However, the use of another aggregation-prone protein as a control would have required a substantial amount of time and work. We intend to consider this in a follow-up project.

12- Minor: Please correct "meduim" in Fig. 2I. Please check for typos throughout.

Re: We have carefully reviewed the entire manuscript to correct all typographical and grammatical errors.

Reviewer #4

1- Although the authors provide compelling data that LIPA A-syn inclusions disrupt share some biochemical traits with Lewy bodies, the morphological contrast between the two is not discussed in much detail. With the exception of some of the HEK cells in Fig 1, the LIPA inclusions tend to be small and numerous (and lacking obvious fibrils) whereas Lewy bodies are typically large with 1-2 present per neuron. The resemblance with Lewy bodies should therefore be toned down in this context.

Re: As per the reviewer's suggestion, we toned down the statement regarding the resemblance between the LIPA-α-syn aggregates and authentic LBs.

2- The in vivo data in Figs. 4 and 5 are highly interesting but omits some quantitative details. For example, what was the distrubution of LIPA A-syn aggregates and what % of TH neurons developed them? Was there spread detected in areas outside of the midbrain and were aggregates induced in any non-neuronal cells?

Re: We performed a quantitative analysis which revealed that 80% of the TH+ midbrain neurons express the LIPA-α-syn construct and exhibit LIPA-α-syn aggregates in the illuminated condition. Moreover, we show that the LIPA-α-syn is mainly neuronal, as 98% of the mCherry+ cells stained positive for NeuN. These data have now been added to the main text (page 13) as well as to Figure S8C-G.

3- In several places in the manuscript, the authors refer to "monomeric A-syn-GFP" (e.g. page 10, 2nd paragraph) when describing recruited A-syn, which may be (but more likely not) in the monomeric form. This should be modified to simply "A-syn-GFP" to avoid confusion.

Re: As per the reviewer's suggestion, we changed "monomeric A-syn-GFP" by "A-syn-GFP" in the main text (page 10 and 11, and figure legends - pages 54, 62, 65).

4- Image in Fig 5B is very hard to make out and was not sufficient for this reviewer to evaluate. Please include one with higher contrast and resolution.

Re: We have replaced the image of Figure 5B.

5- Figures 5G-I: the control graphs from Fig S8 should be included here in the main figure as well. According to the color scale provided, LIPA+light appears to increase the amplitude of activity within the first 100 sec (Fig 5F middle panel). If that is the case, this should be mentioned in the text.

Re: As per the reviewer's suggestion, we moved the control graphs from the supplemental Figure S8 to the main Figure 5. With regard to changes in the amplitude (Fig 5F middle panel), this was observed only in one animal and our quantification across all animals did not show any significant changes.
